# High-Frequency, Short-Session Exercise Decreases Anxiety and Depression in Individuals with Type 2 Diabetes Mellitus: A Systematic Review and Meta-Analysis of Randomized Controlled Trials

**DOI:** 10.3390/bs16010015

**Published:** 2025-12-21

**Authors:** Yexi Tang, Yifan Zhang, Hao Su, Yuanyuan Lv, Meirong Sun, Laikang Yu

**Affiliations:** 1 Beijing Key Laboratory of Sports Performance and Skill Assessment, Beijing 100084, China; helios0529@126.com; 2 Department of Strength and Conditioning Assessment and Monitoring, Beijing Sport University, Beijing 100084, China; 18738314013@163.com (Y.Z.); 15662755237@163.com (H.S.); 3 China Institute of Sport and Health Science, Beijing Sport University, Beijing 100084, China; sunflowerlyy@bsu.edu.cn; 4 School of Psychology, Beijing Sport University, Beijing 100084, China; 5 Key Laboratory of Exercise and Physical Fitness (Beijing Sport University), Ministry of Education, Beijing 100084, China; 6 Laboratory of Sports Stress and Adaptation of General Administration of Sport, Beijing 100084, China

**Keywords:** exercise, anxiety, depression, type 2 diabetes mellitus

## Abstract

This study aimed to evaluate the effects of exercise on anxiety and depression in individuals with type 2 diabetics mellitus (T2DM) and to determine the optimal exercise prescription for this population. A comprehensive search of PubMed, Web of Science, EBSCO, Cochrane Library, and Scopus databases was conducted through 13 May 2025. Pooled analyses were performed using standardized mean difference (SMD) and 95% confidence interval. Exercise significantly alleviated anxiety (SMD, −0.66, *p* < 0.00001) and depression (SMD, −0.55, *p* < 0.0001) in individuals with T2DM. Subgroup analyses revealed that aerobic exercise (anxiety, SMD, −0.68, *p* < 0.00001; depression, SMD, −0.63, *p* = 0.005) and interventions performed ≥3 times per week (anxiety, SMD, −0.72, *p* < 0.00001; depression, SMD, −0.72, *p* < 0.00001), lasting < 60 min per session (anxiety, SMD, −0.78, *p* < 0.00001; depression, SMD, −0.67, *p* < 0.00001), and accumulating ≤ 180 min per week (anxiety, SMD, −0.60, *p* < 0.00001; depression, SMD, −0.60, *p* = 0.0008) were most effective in alleviating anxiety and depression. While the findings of this meta-analysis suggest that engaging in exercise at a frequency of at least 3 times per week, with each session lasting less than 60 min (accumulating to approximately 180 min per week), may be associated with greater psychological improvements, these recommendations should be interpreted cautiously due to variability in study quality, intervention types, and sample characteristics.

## 1. Introduction

Type 2 diabetes mellitus (T2DM) is a prevalent metabolic disorder, with the global prevalence of diabetes estimated at 9.3% in 2019, projected to increase to 10.2% by 2030, and to reach 10.9% by 2045 (AlFalasi et al., 2024). T2DM accounts for over 90% of all diabetes cases worldwide. Importantly, the prevalence of anxiety and depression is markedly higher among individuals with T2DM than in the general population. For instance, a cross-sectional study of Chinese patients with T2DM reported that 56.1% exhibited anxiety symptoms, while 43.6% presented with depressive symptoms (Sun et al., 2016).

Clinically, the early manifestations of T2DM are often subtle. However, as the disease progresses, patients may develop polydipsia, polyuria, fatigue, weight loss, and blurred vision. Persistent hyperglycemia can also lead to various complications (Heuer, 2014), many of which are closely associated with anxiety and depression. Kodakandla et al. (2016) demonstrated significant associations between psychological distress and diabetic neuropathy, diabetic retinopathy, and perceived stress. Anxiety and depression further diminish patients’ quality of life (Alwhaibi, 2024; de Ornelas Maia et al., 2013; Eren et al., 2008; Owens-Gary et al., 2019; Albai et al., 2024; Abualhamael et al., 2024). Anxiety is typically characterized by excessive worry, restlessness, low mood, fatigue, and difficulty concentrating (Adwas et al., 2019), which collectively impair daily functioning, occupational performance, and interpersonal relationships. Depressive symptoms, including persistent sadness, hopelessness, anhedonia, appetite changes, and sleep disturbances, often compromise self-care and social interactions (Kendler, 2016). Moreover, anxiety and depression negatively affect treatment adherence, as low mood may lead patients to neglect medical advice or refuse therapy, thereby undermining long-term disease management (Azad et al., 2014).

The occurrence of anxiety and depression in T2DM is influenced by multiple factors, including gender, sleep quality, and lifestyle. Among lifestyle factors, exercise is particularly associated with improvements in psychological well-being (Ali, 2013). Rebello et al. (2022) demonstrated that exercise alleviates anxiety and depression in individuals without diabetes by enhancing self-esteem, improving body image, increasing serotonin release, and regulating stress response. Similar benefits have been reported in individuals with T2DM (Dhasaradharaman et al., 2018; Boughdady et al., 2023; Youssef, 2019). For example, a randomized controlled trial (RCT) with 80 participants found that four months of exercise intervention significantly reduced anxiety and depression symptoms while improving quality of life (Abbas et al., 2011). Beyond direct emotional relief, exercise may enhance overall mental health by reducing social barriers, alleviating insomnia and physical discomfort, and fostering greater self-efficacy in disease management (Sardar et al., 2014). Nevertheless, evidence is not unequivocal. Reid et al. (2010) observed better mental health outcomes in a non-exercising control group compared with an exercising group, while Van der Heijden et al. (2013) concluded that the psychological benefits of exercise in T2DM remain inconclusive, as some studies report non-significant effects.

Several meta-analyses have investigated the influence of exercise on anxiety and depression in individuals with T2DM. Arsh et al. (2023) suggested that exercise can effectively alleviate depressive symptoms in this population; however, some studies included in that review allowed exercise interventions in the control groups, potentially confounding the findings. Van der Heijden et al. (2013) also highlighted that much of the current research relies on observational designs and lacks robust RCTs to provide more definitive evidence.

Accordingly, the present study synthesizes evidence from high-quality RCTs to evaluate the effects of exercise on anxiety and depression in individuals with T2DM. The objective is to determine the optimal exercise regimen for mitigating anxiety and depression in this population.

## 2. Materials and Methods

This systematic review and meta-analysis were conducted in accordance with the Preferred Reporting Items for Systematic Reviews and Meta-Analyses (PRISMA) guidelines (Page et al., 2021). The study protocol was preregistered in PROSPERO (registration number: CRD420251065667).

### 2.1. Search Strategy

A comprehensive literature search was undertaken across five electronic databases: PubMed, Web of Science, Cochrane Library, Scopus, and EBSCO. The search covered studies published from database inception to 13 May 2025. Relevant keywords and Medical Subject Headings (MeSH) related to exercise, diabetes, anxiety, and depression were applied (Table S1). Additionally, the reference lists of all included studies were manually screened to identify further eligible studies. Two authors (Y.T. and Y.Z.) independently performed the search and study selection. Any discrepancies were resolved through discussion with a third author (L.Y.) until consensus was achieved.

### 2.2. Inclusion and Exclusion Criteria

The Population, Intervention, Comparison, Outcome, Study design (PICOS) framework was used to define the inclusion criteria. (1) Population: participants diagnosed with T2DM; (2) Intervention: participants randomly assigned to either the intervention group or control group; (3) Comparison: studies that measured anxiety or depression before and after the intervention; (4) Outcome: the primary outcome was anxiety and depression; and (5) Study design: RCT design.

Exclusion criteria were: (1) publications in languages other than English; (2) review articles or conference abstracts; (3) studies involving animal models; (4) lack of available full-text; and (5) outcomes that could not be transformed into mean and standard deviation (SD) values.

### 2.3. Data Extraction

Two authors (Y.T. and Y.Z.) independently extracted data using a standardized form, with discrepancies resolved by consensus with a third author (L.Y.). Extracted data included: (1) study characteristics (first author, publication year, sample size); (2) intervention details (intervention type, duration, frequency, session duration, and weekly time); (3) participant characteristics (age and body mass index [BMI]); and (4) outcomes (pre- to post-intervention changes in anxiety or depression scales scores).

### 2.4. Quality Assessment

Risk of bias for the included studies was independently evaluated by two authors (Y.T. and Y.Z.) using the Cochrane Risk of Bias (RoB) tool (Qiu et al., 2024). Six domains were assessed: random sequence generation, allocation concealment, blinding, missing outcome data, selective reporting, and other biases. Each domain was rated as “low risk”, “high risk”, or “unclear risk” (Zhou et al., 2024). Discrepancies were resolved by discussion with a third author (L.Y.).

### 2.5. Statistical Analysis

For each study, changes in mean and SD values for anxiety and depression scales scores were calculated. When only standard error (SE) or 95% confidence intervals (CIs) were reported, SD values were estimated (Wu et al., 2024; Du et al., 2024). Standardized mean difference (SMD) and 95% CI were used to estimate the effects of exercise on anxiety and depression in individuals with T2DM. Heterogeneity was assessed using the I^2^ statistic, with I^2^ > 50% indicating substantial heterogeneity. Data were pooled using fixed effects models or random effects models when I^2^ < 50% or I^2^ ≥ 50%, respectively. In such cases, meta-regression, subgroup, and sensitivity analyses were performed to explore potential sources of variability (Higgins & Thompson, 2002).

Subgroup analyses were stratified by intervention type (aerobic exercise, multicomponent training), frequency (<3 times per week vs. ≥3 times per week), session duration (<60 min vs. ≥60 min), and weekly time (≤180 min vs. >180 min). Forest plots were generated using RevMan 5.4 software, while Egger’s test, sensitivity analysis, meta-regression, and funnel plots were performed using Stata 18. A *p*-value < 0.05 was considered statistically significant.

## 3. Results

### 3.1. Study Selection

**Figure 1:**
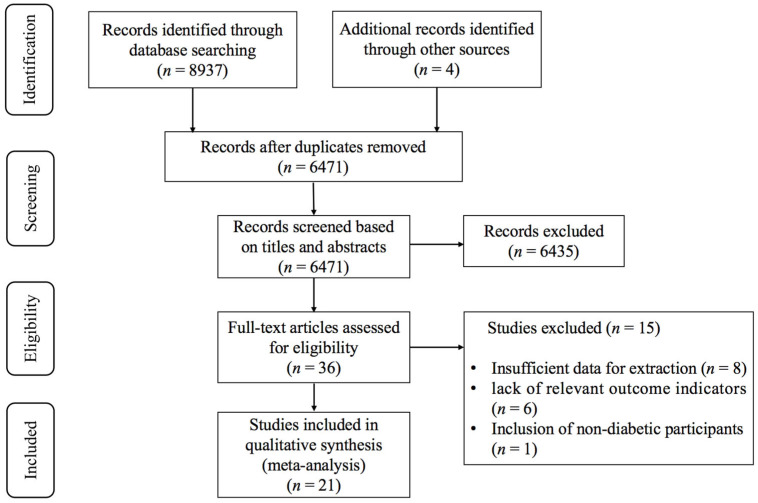
PRISMA flowchart of study selection.

As illustrated in Figure 1, the initial database search identified 8937 records, with an additional 4 articles via other sources. After removing duplicates, 6471 articles remained. Screening of titles and abstracts excluded 6435 articles that did not meet the inclusion criteria. The remaining 36 articles underwent full-text assessment, of which 15 were excluded for the following reasons: (1) insufficient data for extraction (n = 8); (2) lack of relevant outcome indicators (n = 6); (3) inclusion of individuals without diabetes (n = 1). Ultimately, 21 studies (Abdelbasset et al., 2020; Aikens et al., 1997; Aylin et al., 2009; Donyaei et al., 2024; Duruturk & Özköslü, 2019; Gilani & Feizabad, 2019; Kraiwong et al., 2021; Leehey et al., 2016; Lincoln et al., 2011; Maharaj & Nuhu, 2023; Martínez-Velilla et al., 2021; Osama & Shehab, 2015; Pibernik-Okanović et al., 2015; Piette et al., 2011; Saiiari et al., 2011; Sardar et al., 2014; Schneider et al., 2016; Snel et al., 2012; Yadav et al., 2021; Yucel & Uysal, 2016; Zheng et al., 2015) were included in the meta-analysis.

### 3.2. Characteristics of the Included Studies

Table S2 summarizes the key characteristics of the included studies. Five were conducted in the United States (Aikens et al., 1997; Leehey et al., 2016; Lincoln et al., 2011; Piette et al., 2011; Schneider et al., 2016), four in Iran (Donyaei et al., 2024; Gilani & Feizabad, 2019; Saiiari et al., 2011; Sardar et al., 2014), three in Turkey (Aylin et al., 2009; Duruturk & Özköslü, 2019; Yucel & Uysal, 2016), two in Saudi Arabia (Abdelbasset et al., 2020; Osama & Shehab, 2015), and one each in Thailand (Kraiwong et al., 2021), South Africa (Maharaj & Nuhu, 2023), Spain (Martínez-Velilla et al., 2021), Croatia (Pibernik-Okanović et al., 2015), Netherlands (Snel et al., 2012), India (Yadav et al., 2021), and China (Zheng et al., 2015). In total, the studies enrolled 1435 participants with confirmed T2DM, comprising 725 participants in intervention groups and 710 participants in control groups.

Of these, 14 studies employed multicomponent training (Abdelbasset et al., 2020; Aikens et al., 1997; Aylin et al., 2009; Donyaei et al., 2024; Duruturk & Özköslü, 2019; Kraiwong et al., 2021; Leehey et al., 2016; Martínez-Velilla et al., 2021; Pibernik-Okanović et al., 2015; Piette et al., 2011; Schneider et al., 2016; Yadav et al., 2021; Yucel & Uysal, 2016; Zheng et al., 2015), 6 studies examined aerobic exercise (Gilani & Feizabad, 2019; Maharaj & Nuhu, 2023; Osama & Shehab, 2015; Saiiari et al., 2011; Sardar et al., 2014; Snel et al., 2012), and 1 study focused on resistance exercise (Lincoln et al., 2011). Intervention durations ranged from 6 to 48 weeks (mean: 15.18 weeks), with frequencies from 1 to 12 sessions per week (mean: 3.65 sessions). Session lengths ranged from 20 and 90 min (mean: 54.52 min), while weekly durations ranged from 60 min to over 180 min (mean: 162.74 min). One study did not report detailed intervention parameters (Piette et al., 2011).

### 3.3. Meta-Analysis

**Figure 2:**
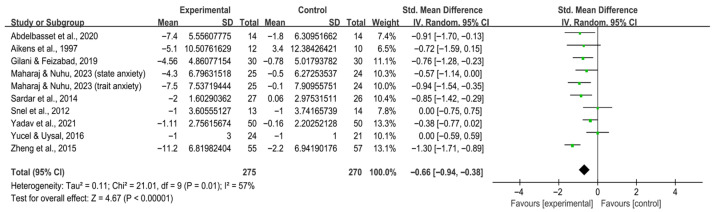
Meta-analysis results of the effect of exercise on anxiety in individuals with T2DM (Abdelbasset et al., 2020; Aikens et al., 1997; Gilani & Feizabad, 2019; Maharaj & Nuhu, 2023; Sardar et al., 2014; Snel et al., 2012; Yadav et al., 2021; Yucel & Uysal, 2016; Zheng et al., 2015).

**Figure 3:**
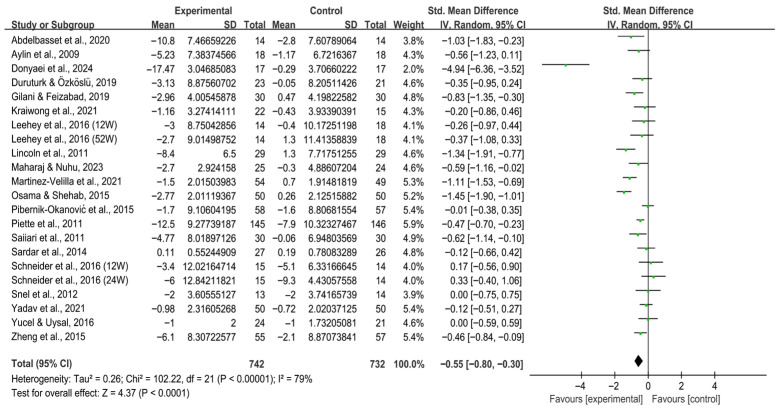
Meta-analysis results of the effect of exercise on depression in individuals with T2DM (Abdelbasset et al., 2020; Aylin et al., 2009; Donyaei et al., 2024; Duruturk & Özköslü, 2019; Gilani & Feizabad, 2019; Kraiwong et al., 2021; Leehey et al., 2016; Lincoln et al., 2011; Maharaj & Nuhu, 2023; Martínez-Velilla et al., 2021; Osama & Shehab, 2015; Pibernik-Okanović et al., 2015; Piette et al., 2011; Saiiari et al., 2011; Sardar et al., 2014; Schneider et al., 2016; Snel et al., 2012; Yadav et al., 2021; Yucel & Uysal, 2016; Zheng et al., 2015). 12W, intervention duration of 12 weeks; 24W, intervention duration of 24 weeks; 52W, intervention duration of 52 weeks.

Exercise significantly reduced anxiety [SMD, −0.66 (95% CI, −0.94 to −0.38), *p* < 0.00001, I^2^ = 57%, Figure 2] and depression [SMD, −0.55 (95% CI, −0.80 to −0.30), *p* < 0.0001, I^2^ = 79%, Figure 3] in individuals with T2DM.

### 3.4. Subgroup Analysis

#### 3.4.1. Anxiety

**Figure 4:**
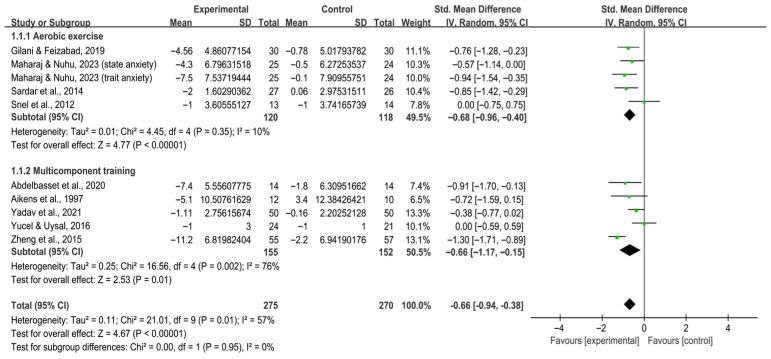
Meta-analysis results of the effect of types of intervention on anxiety in individuals with T2DM (Abdelbasset et al., 2020; Aikens et al., 1997; Gilani & Feizabad, 2019; Maharaj & Nuhu, 2023; Sardar et al., 2014; Snel et al., 2012; Yadav et al., 2021; Yucel & Uysal, 2016; Zheng et al., 2015).

Subgroup analysis by intervention type revealed that aerobic exercise [SMD, −0.68 (95% CI, −0.96 to −0.40), *p* < 0.00001, I^2^ = 10%] and multicomponent training [SMD, −0.66 (95% CI, −1.17 to −0.15), *p* = 0.01, I^2^ = 76%, Figure 4] both statistically alleviated anxiety in individuals with T2DM.

**Figure 5:**
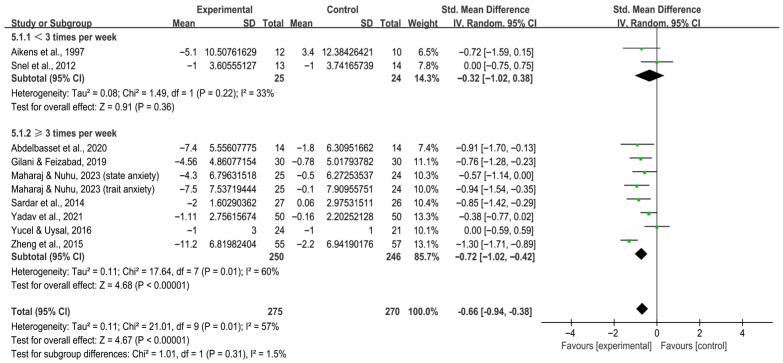
Meta-analysis results of the effect of frequency of intervention on anxiety in individuals with T2DM (Abdelbasset et al., 2020; Aikens et al., 1997; Gilani & Feizabad, 2019; Maharaj & Nuhu, 2023; Sardar et al., 2014; Snel et al., 2012; Yadav et al., 2021; Yucel & Uysal, 2016; Zheng et al., 2015).

When stratified by frequency, interventions conducted ≥3 times per week significantly alleviated anxiety in individuals with T2DM [SMD, −0.72 (95% CI, −1.02 to −0.42), *p* < 0.00001, I^2^ = 60%], whereas <3 times per week did not [SMD, −0.32 (95% CI, −1.02 to 0.38), *p* = 0.36, I^2^ = 33%, Figure 5].

**Figure 6:**
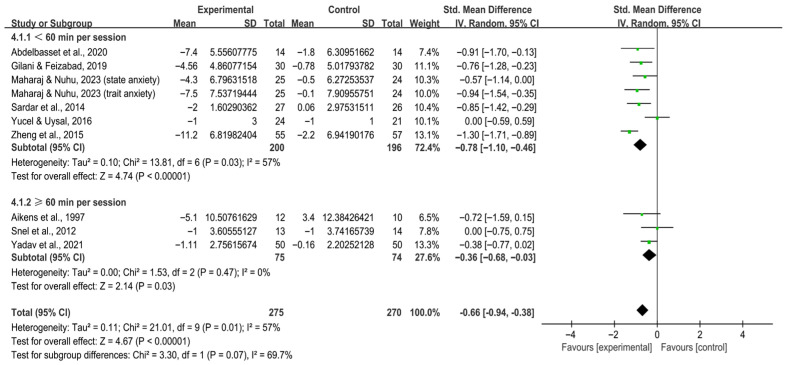
Meta-analysis results of the effect of duration of intervention per session on anxiety in individuals with T2DM (Abdelbasset et al., 2020; Aikens et al., 1997; Gilani & Feizabad, 2019; Maharaj & Nuhu, 2023; Sardar et al., 2014; Snel et al., 2012; Yadav et al., 2021; Yucel & Uysal, 2016; Zheng et al., 2015).

Subgroup analysis by session duration revealed that both < 60 min per session [SMD, −0.78 (95% CI, −1.10 to −0.46), *p* < 0.00001, I^2^ = 57%] and ≥ 60 min per session [SMD, −0.36 (95% CI, −0.68 to −0.03), *p* = 0.03, I^2^ = 0%, Figure 6] significantly alleviated anxiety in individuals with T2DM.

**Figure 7:**
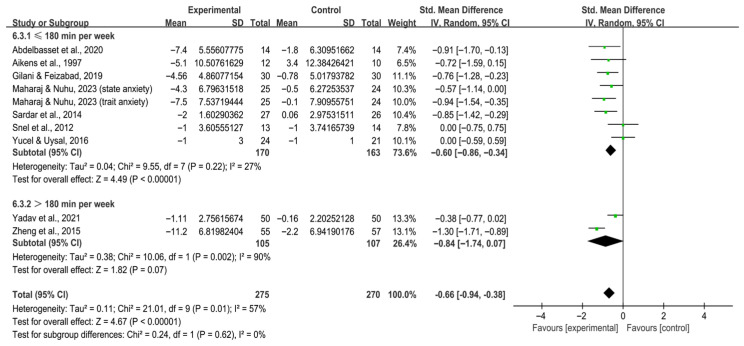
Meta-analysis results of the effect of duration of intervention per week on anxiety in individuals with T2DM (Abdelbasset et al., 2020; Aikens et al., 1997; Gilani & Feizabad, 2019; Maharaj & Nuhu, 2023; Sardar et al., 2014; Snel et al., 2012; Yadav et al., 2021; Yucel & Uysal, 2016; Zheng et al., 2015).

Stratification by weekly time showed that ≤180 min per week significantly alleviated anxiety in individuals with T2DM [SMD, −0.60 (95% CI, −0.86 to −0.34), *p* < 0.00001, I^2^ = 27%], whereas >180 min per week did not [SMD, −0.84 (95% CI, −1.74 to 0.07), *p* = 0.07, I^2^ = 90%, Figure 7].

#### 3.4.2. Depression

**Figure 8:**
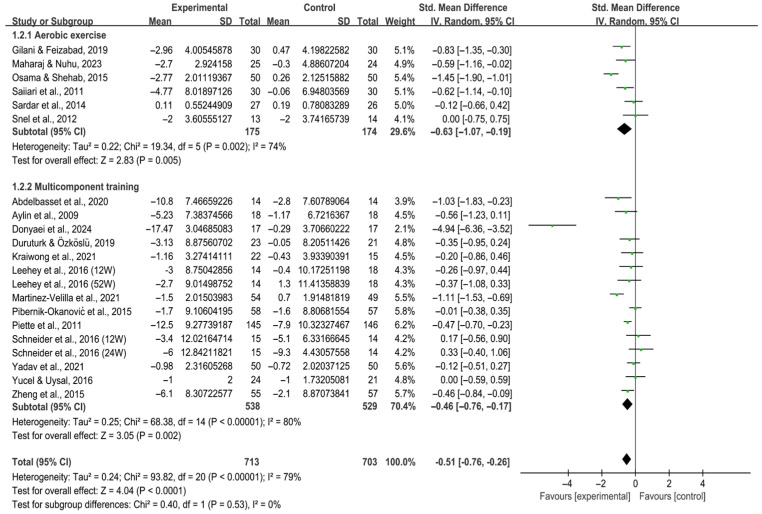
Meta-analysis results of the effect of types of intervention on depression in individuals with T2DM (Abdelbasset et al., 2020; Aylin et al., 2009; Donyaei et al., 2024; Duruturk & Özköslü, 2019; Gilani & Feizabad, 2019; Kraiwong et al., 2021; Leehey et al., 2016; Maharaj & Nuhu, 2023; Martínez-Velilla et al., 2021; Osama & Shehab, 2015; Pibernik-Okanović et al., 2015; Piette et al., 2011; Saiiari et al., 2011; Sardar et al., 2014; Schneider et al., 2016; Snel et al., 2012; Yadav et al., 2021; Yucel & Uysal, 2016; Zheng et al., 2015). 12W, intervention duration of 12 weeks; 24W, intervention duration of 24 weeks; 52W, intervention duration of 52 weeks.

Both aerobic exercise [SMD, −0.63 (95% CI, −1.07 to −0.19), *p* = 0.005, I^2^ = 74%] and multicomponent training [SMD, −0.46 (95% CI, −0.76 to −0.17), *p* = 0.002, I^2^ = 80%, Figure 8] significantly alleviated depression in individuals with T2DM, with greater effects observed in aerobic exercise.

**Figure 9:**
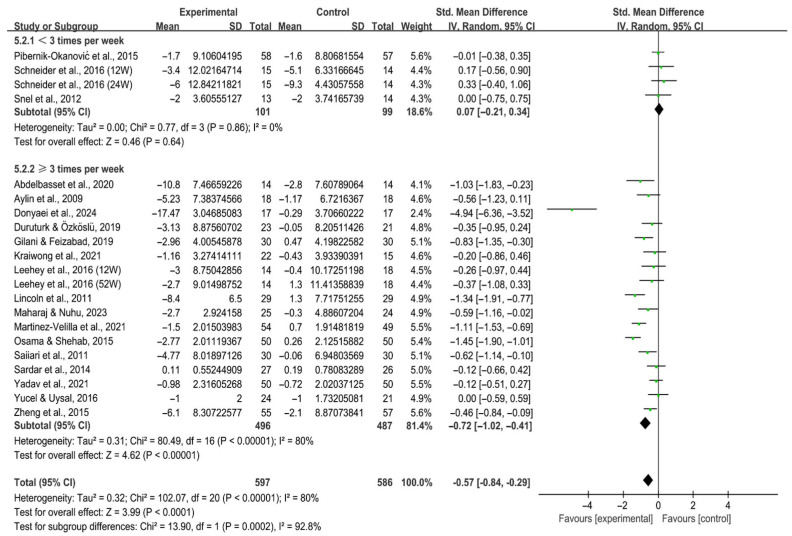
Meta-analysis results of the effect of frequency of intervention on depression in individuals with T2DM (Abdelbasset et al., 2020; Aylin et al., 2009; Donyaei et al., 2024; Duruturk & Özköslü, 2019; Gilani & Feizabad, 2019; Kraiwong et al., 2021; Leehey et al., 2016; Lincoln et al., 2011; Maharaj & Nuhu, 2023; Martínez-Velilla et al., 2021; Osama & Shehab, 2015; Pibernik-Okanović et al., 2015; Saiiari et al., 2011; Sardar et al., 2014; Schneider et al., 2016; Snel et al., 2012; Yadav et al., 2021; Yucel & Uysal, 2016; Zheng et al., 2015). 12W, intervention duration of 12 weeks; 24W, intervention duration of 24 weeks; 52W, intervention duration of 52 weeks.

Interventions performed ≥3 times per week [SMD, −0.72 (95% CI, −1.02 to −0.41), *p* < 0.00001, I^2^ = 80%] significantly alleviated depression in individuals with T2DM, while those < 3 times per week did not [SMD, 0.07 (95% CI, −0.21 to 0.34), *p* = 0.64, I^2^ = 0%, Figure 9].

**Figure 10:**
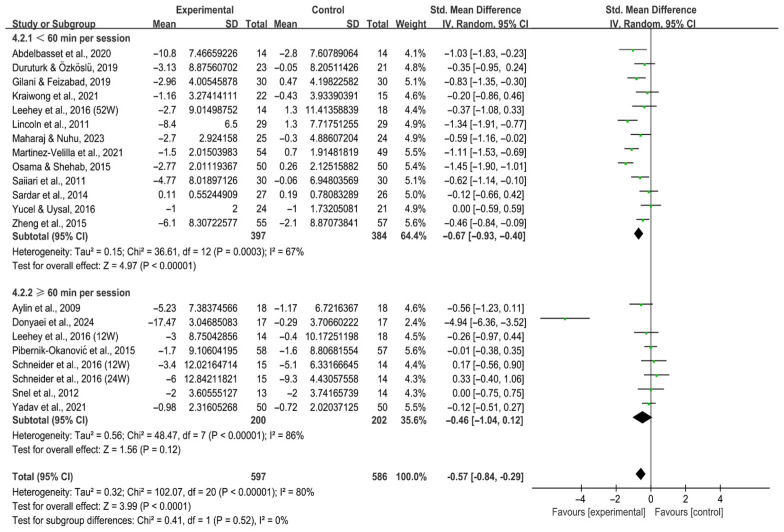
Meta-analysis results of the effect of duration of intervention per session on depression in individuals with T2DM (Abdelbasset et al., 2020; Aylin et al., 2009; Donyaei et al., 2024; Duruturk & Özköslü, 2019; Gilani & Feizabad, 2019; Kraiwong et al., 2021; Leehey et al., 2016; Lincoln et al., 2011; Maharaj & Nuhu, 2023; Martínez-Velilla et al., 2021; Osama & Shehab, 2015; Pibernik-Okanović et al., 2015; Saiiari et al., 2011; Sardar et al., 2014; Schneider et al., 2016; Snel et al., 2012; Yadav et al., 2021; Yucel & Uysal, 2016; Zheng et al., 2015). 12W, intervention duration of 12 weeks; 24W, intervention duration of 24 weeks; 52W, intervention duration of 52 weeks.

For session duration, interventions < 60 min per session significantly alleviated depression in individuals with T2DM [SMD, −0.67 (95% CI, −0.93 to −0.40), *p* < 0.00001, I^2^ = 67%], whereas ≥60 min per session showed no significant effect [SMD, −0.46 (95% CI, −1.04 to 0.12), *p* = 0.12, I^2^ = 86%, Figure 10].

**Figure 11:**
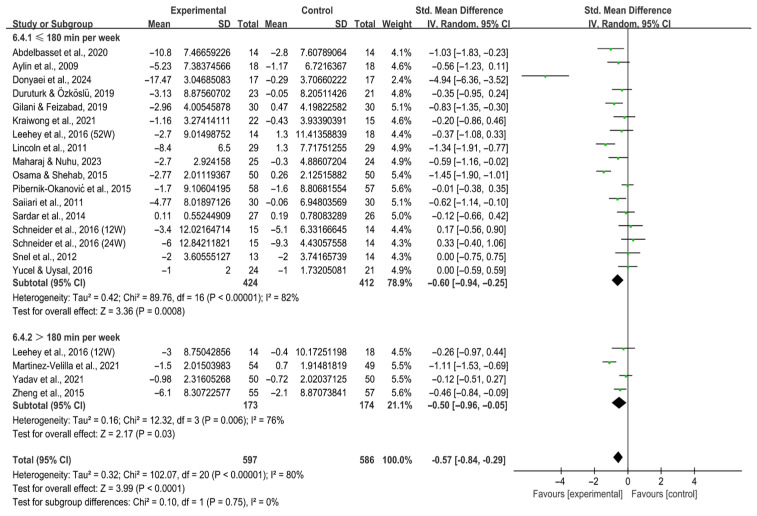
Meta-analysis results of the effect of duration of intervention per week on depression in individuals with T2DM (Abdelbasset et al., 2020; Aylin et al., 2009; Donyaei et al., 2024; Duruturk & Özköslü, 2019; Gilani & Feizabad, 2019; Kraiwong et al., 2021; Leehey et al., 2016; Lincoln et al., 2011; Maharaj & Nuhu, 2023; Martínez-Velilla et al., 2021; Osama & Shehab, 2015; Pibernik-Okanović et al., 2015; Saiiari et al., 2011; Sardar et al., 2014; Schneider et al., 2016; Snel et al., 2012; Yadav et al., 2021; Yucel & Uysal, 2016; Zheng et al., 2015). 12W, intervention duration of 12 weeks; 24W, intervention duration of 24 weeks; 52W, intervention duration of 52 weeks.

Analysis by weekly time revealed that both ≤180 min per week [SMD, −0.60 (95% CI, −0.94 to −0.25), *p* = 0.0008, I^2^ = 82%] and >180 min per week [SMD, −0.50 (95% CI, −0.96 to −0.05), *p* = 0.03, I^2^ = 76%, Figure 11] significantly alleviated depression in individuals with T2DM, with lower volumes produced larger effects.

### 3.5. Meta-Regression

As presented in Table S3, the meta-regression analyses revealed no statistically significant associations between effect size and intervention type (anxiety: *p* = 0.978; depression: *p* = 0.773), frequency (anxiety: *p* = 0.356; depression: *p* = 0.083), session duration (anxiety: *p* = 0.175; depression: *p* = 0.614), or weekly time (anxiety: *p* = 0.490; depression: *p* = 0.828).

### 3.6. Risk of Bias

As summarized in Figure S1, the included study quality was categorized as high, moderate, or low via the criteria (Zhang et al., 2025a). Specifically: (1) trials with high risk of bias in randomization or allocation concealment were rated low quality; (2) trials with low risk in both domains and all others rated as low or unclear were considered high quality; and (3) all others were rated as moderate quality. Among the 21 studies, 4 studies (Donyaei et al., 2024; Duruturk & Özköslü, 2019; Piette et al., 2011; Schneider et al., 2016) were rated as high quality, while 17 studies (Sardar et al., 2014; Abdelbasset et al., 2020; Aikens et al., 1997; Aylin et al., 2009; Gilani & Feizabad, 2019; Kraiwong et al., 2021; Leehey et al., 2016; Lincoln et al., 2011; Maharaj & Nuhu, 2023; Martínez-Velilla et al., 2021; Osama & Shehab, 2015; Pibernik-Okanović et al., 2015; Saiiari et al., 2011; Snel et al., 2012; Yadav et al., 2021; Yucel & Uysal, 2016; Zheng et al., 2015) while as moderate quality.

### 3.7. Publication Bias

Funnel plots for both anxiety and depression outcomes showed no substantial asymmetry (Figures S2 and S3). Egger’s test confirmed the absence of significant publication bias for anxiety (t = 0.66, *p* = 0.527, Table S4) and depression (t = −0.84, *p* = 0.409, Table S4).

### 3.8. Sensitivity Analysis

Sensitivity analyses demonstrated that the pooled effects of exercise on anxiety (Figure S4) and depression (Figure S5) remained robust, with no single study exerting undue influence on the overall results.

## 4. Discussion

### 4.1. Effects of Exercise on Anxiety in Individuals with T2DM

The beneficial effects of exercise on anxiety in individuals with T2DM are consistent with previous findings. Van der Heijden et al. (2013) indicated that exercise led to significant improvements in anxiety assessments. Similarly, a study on elderly patients with T2DM showed that exercise simultaneously enhanced physical fitness while reducing anxiety and depression levels (Boughdady et al., 2023).

Exercise alleviates anxiety in individuals with T2DM through multiple physiological and psychosocial mechanisms. Physiologically, exercise modulates neuroendocrine function by increasing brain-derived neurotrophic factor (BDNF) levels, thereby enhancing mood and cognition (Riyahi Malayeri et al., 2021; Rozanska et al., 2020), while reducing cortisol levels that trigger stress responses (Obaya et al., 2021). It also promotes the release of endorphins associated with well-being (Enayatjazi et al., 2015), directly improving mood. In addition, exercise reduces levels of inflammatory mediators such as interleukin-6 (IL-6) and tumor necrosis factor-alpha (TNF-α), indirectly alleviating anxiety by improving inflammatory status (Liu et al., 2015), which are closely associated with anxiety and depression (Peng et al., 2024). On a psychosocial level, exercise enhances patients’ self-efficacy and social functioning while strengthening stress-coping abilities (Siregar et al., 2018). One study demonstrated that aerobic exercise improves mental health, anxiety, and insomnia (Sardar et al., 2014). Exercise also improves cognitive function and strengthens emotional regulation (Praet & Van Loon, 2009). Together, these mechanisms support the notion that exercise is an effective non-pharmacological intervention for alleviating anxiety in individuals with T2DM.

Although some studies support the benefits of exercise for anxiety in this population, other research reports either insignificant or controversial effects. Reid et al. (2010) found that the non-exercising control group showed superior mental health outcomes compared with aerobic or resistance exercise groups. Such contradictions may be related to individual differences, research rigor, exercise type and intensity, as well as psychological disorders and sociocultural factors (Gebeyehu et al., 2022).

### 4.2. Effects of Different Exercise Modalities on Anxiety in Individuals with T2DM

The results of subgroup analysis indicated that both aerobic exercise and multicomponent training effectively alleviate anxiety in individuals with T2DM (Sardar et al., 2014; Gebeyehu et al., 2022), though through different mechanisms. Aerobic exercise directly relieves anxiety by increasing BDNF and endorphin levels, improving autonomic nervous system function, enhancing insulin sensitivity, and reducing inflammatory factors. Multicomponent training indirectly alleviates anxiety by using exercise variety to enhance psychological engagement and sense of accomplishment, thereby strengthening psychological resilience.

Comparison suggests that aerobic exercise yields superior outcomes compared with multicomponent training. Possible reasons include: (1) aerobic exercise exerts direct neuromodulatory effects on anxiety (Herik et al., 2021); (2) resistance exercise within multicomponent protocols exerts relatively limited influence on mental health, and its improvements in psychological indicators such as anxiety may be overestimated (Reid et al., 2010; Yavari et al., 2012); and (3) patients demonstrate higher tolerance for aerobic exercise, whereas resistance exercise may reduce adherence due to muscle fatigue or discomfort, thereby affecting overall outcomes (Van der Heijden et al., 2013). Furthermore, some multicomponent interventions were of low intensity. While some research suggests low-intensity exercise may alleviate anxiety in individuals with diabetes (Ahmad & Ali, 2020; Ji et al., 2022), Colberg (2012) indicated that moderate-to-high-intensity exercise is more effective in improving glycemic control, which may also have diminished the efficacy of multicomponent training. However, few studies directly compare the anxiety-alleviating effects of these two training types in individuals with T2DM. Moreover, the lack of standardized multicomponent training protocols reduces comparability, potentially affecting consistency of results.

Given that both aerobic exercise and multicomponent training alleviated anxiety in individuals with T2DM, this study further stratified subgroup analyses by frequency, session duration, and weekly time. Results revealed that exercising ≥ 3 times per week, with sessions < 60 min and a total weekly duration ≤ 180 min, significantly reduced anxiety.

Multiple studies indicate that frequency is strongly associated with improvements in anxiety (Gilani & Feizabad, 2019; Boughdady et al., 2023; Lestari et al., 2024). Aerobic exercise 3–5 times per week significantly alleviates anxiety in individuals with T2DM. The American College of Sports Medicine (ACSM) guidelines also recommend at least 150 min of moderate-intensity aerobic exercise weekly, distributed over 3–5 days (Nelson et al., 2007). Furthermore, one study reported that exercising ≥ 3 times per week significantly alleviates anxiety in breast cancer survivors (Zhang et al., 2025b). This may be explained by frequent exercise facilitating physiological adaptation and establishing consistent habits, thereby alleviating anxiety. Van der Heijden et al. (2013) similarly found that aerobic exercise performed three or more times per week yielded significant effects despite inconsistent overall results. A cross-sectional study further reported that exercising 1–2 times per week was associated with reduced anxiety risk, but ≥3 times per week produced greater benefits (Hallgren et al., 2020). Therefore, intervention strategies should prioritize the benefits of exercising at least three times weekly.

With respect to session duration, subgroup analysis showed that although ≥60 min per session significantly alleviated anxiety, effects are more pronounced for sessions < 60 min. The impact of session duration remains controversial. However, most studies reporting significant anxiety-relieving effects limited session durations to <60 min (Gilani & Feizabad, 2019; Oberlin et al., 2014; Perkins et al., 2023). Adams (2013) indicated that a single 60 min high-intensity session may trigger a hyperglycemic response within one hour post-meal, while Eshghi et al. (2017) observed continued elevations in blood glucose and reduced insulin sensitivity within 60 min post-exercise in individuals with T2DM. Considering that physical fitness in this population is often compromised (Bagbila et al., 2022; Seyam & Seyam, 2019), excessively long sessions may negate benefits due to insufficient metabolic adaptation or excessive fatigue. Eriksen et al. (2007) revealed that three 10 min sessions per day improved glycemic control more effectively than a single 30 min session, as multiple short bouts sustain energy expenditure and metabolic activity. This finding further supports the advantage of shorter but more frequent sessions.

Lestari et al. (2024) reported that exercising three times per week for 60 min per session (totaling 180 min) significantly reduces blood glucose levels, whereas daily 30 min sessions (totaling 210 min) did not. This aligns with our results: anxiety decreased significantly when weekly duration was ≤180 min, while exceeding 180 min yielded no significant improvements. Considering that upper and lower limb strength in individuals with T2DM decreased by 56.7% and 80%, respectively (Bagbila et al., 2022), excessively long exercise may induce fatigue responses that undermine effectiveness. Thus, exercise exceeding 180 min per week may be suboptimal. Collectively, the evidence suggests that exercise following a “high-frequency, short-duration” pattern, sessions < 60 min, ≥3 times per week, optimally alleviates anxiety in individuals with T2DM.

### 4.3. Effects of Exercise on Depression in Individuals with T2DM

Abbas et al. (2011) indicated that both aerobic and resistance exercise exert positive effects on depression. Rebello et al. (2022) suggested that exercise reduces chronic inflammation by decreasing visceral fat and promoting the secretion of anti-inflammatory cytokines, with inflammation being a key pathological factor in depression. Hong & Barnes (2012) found that exercise increases serum serotonin and norepinephrine levels, mimicking the effects of antidepressants but with fewer side effects, a finding corroborated by Peng et al. (2024). Furthermore, De Groot et al. (2012) demonstrated that combining community-based aerobic exercise with individualized cognitive behavioral therapy (CBT) significantly alleviated depression scores in 66% of patients, highlighting that structured exercise interventions can markedly improve adherence. These findings are consistent with the results of the present study.

Exercise alleviates depression in individuals with T2DM through multiple physiological, psychological, and social mechanisms. Physiologically, exercise enhances insulin sensitivity, improves glucose regulation, and reduces neuropathic damage caused by hyperglycemia (Tomas-Carus et al., 2020). It also reduces visceral fat, improves lipid metabolism, and lowers inflammatory factor levels, thereby mitigating the negative impact of inflammation on the nervous system and contributing to improved glycemic control and metabolic health (Rebello et al., 2022). Moreover, it promotes the secretion of BDNF, thereby enhancing neuroplasticity (Cokar et al., 2022). On the psychosocial level, exercise improves social functioning, enhances self-efficacy and quality of life (Sardar et al., 2014), and consequently reduces depressive symptoms.

However, Van der Heijden et al. (2013) noted conflicting evidence regarding exercise efficacy for depression, with some studies failing to demonstrate significant effects. Possible explanations include intervention design, intensity, and patient motivation. Overtraining may suppress immune function and increase inflammation risk (Rebello et al., 2022). Additionally, some patients may struggle to maintain exercise participation due to motivational or social barriers (Hong & Barnes, 2012). Notably, depressive symptoms themselves often reduce exercise adherence (Rahman et al., 2021), creating a vicious cycle in which “depression leads to reduced exercise, which in turn exacerbates depression”. This dynamic may compromise the effectiveness of exercise interventions.

### 4.4. Effects of Different Exercise Modalities on Depression in Individuals with T2DM

Several studies have shown that combined aerobic and resistance training is more effective than aerobic or resistance training alone for improving glycemic control, mental health, and muscle density (Earnest et al., 2014; Gebeyehu et al., 2022). However, Stephens and Sparks (2015) observed that approximately 15–20% of individuals with T2DM respond poorly to exercise. Further evidence suggests that such patients may experience impaired myocardial contractility, abnormal heart rate and blood pressure regulation, and reduced muscle oxygen utilization during exercise (Nesti et al., 2020). This implies that individuals with T2DM require carefully tailored exercise intensity, and because multicomponent training typically involves higher intensity than aerobic exercise alone, aerobic exercise may produce superior results, as observed in this study.

Subgroup analysis by frequency revealed that exercising ≥ 3 times per week significantly reduced depression, whereas <3 times per week failed to achieve significant improvements in individuals with T2DM, consistent with Youssef (2019). The ACSM also emphasizes “distributed exercise”, recommending 3–5 sessions per week rather than concentrating exercise on fewer days (Nelson et al., 2007). It also acknowledges that all exercise confers benefits: even if individual sessions do not meet target intensity, cumulative benefits can accrue from multiple shorter sessions. Given the reduced exercise tolerance in many individuals with T2DM, clinical practice should prioritize shorter but more frequent sessions to maximize benefits while ensuring safety.

Excessively prolonged exercise sessions may provoke fatigue through neurological, metabolic, inflammatory, and psychological pathways, thereby diminishing benefits. One previous research has confirmed that extended exercise induces interactions between central and peripheral fatigue (Millet & Lepers, 2004). Similarly, long-duration sessions can trigger inflammatory responses and metabolic disturbances, exacerbating fatigue (Nicklas & Brinkley, 2009). Ribeiro et al. (2024) also observed that prolonged exercise significantly increases perceived fatigue, whereas short-duration high-intensity exercise does not. These findings collectively suggest that “excessive duration is a major contributor to fatigue”. Subgroup analysis in this study revealed that interventions < 60 min were effective, whereas those ≥60 min were not, suggesting that fatigue from prolonged exercise may explain these outcomes.

Weekly intervention duration analyses revealed optimal results when total duration was ≤180 min. Although existing studies have not explicitly quantified the relationship between weekly duration and improvements in depressive symptoms, Burooj (2024) reported a negative correlation between exercise duration and diabetes progression. Furthermore, most studies reporting significant improvements in depression restricted weekly exercise to ≤180 min (Youssef, 2019; Gilani & Feizabad, 2019). Considering that excessive exercise may suppress immunity (Rebello et al., 2022), we hypothesize that interventions > 180 min per week may trigger fatigue, thereby diminishing efficacy. Collectively, the subgroup analyses indicate that the most pronounced improvements occur when weekly duration is ≤180 min, session durations are <60 min, and weekly frequency ≥ 3 sessions. This pattern suggests that shorter but more frequent exercise sessions may be more effective for alleviating depression in individuals with T2DM; however, this interpretation should be viewed cautiously and warrants further confirmation in future trials.

### 4.5. Limitations

This study has several limitations. First, all included studies were RCTs of exercise interventions. Due to the inherent nature of exercise interventions, complete blinding is difficult to achieve, potentially introducing subjective bias during quality assessment. Second, some studies did not report exercise intensity in detail, limiting the ability to fully assess how intensity influences anxiety and depression. Third, statistical analyses of exercise intensity and supervision were not performed, restricting the depth of conclusions. Thus, future research with larger sample sizes and higher methodological rigor is needed to validate these findings and strengthen reliability. Additionally, the optimal configuration of aerobic and resistance components within the “high-frequency, short-duration” model remains unclear, warranting further investigation to refine exercise prescriptions. Finally, variations in intervention duration, patients’ baseline health conditions, and specific anxiety or depression triggers may reduce consistency across studies. These factors should be considered when interpreting the conclusions of this study.

## 5. Conclusions

Exercise appears to have beneficial effects in alleviating anxiety and depression in individuals with T2DM. While the findings of this meta-analysis suggest that engaging in exercise at a frequency of at least 3 times per week, with each session lasting less than 60 min (accumulating to approximately 180 min per week), may be associated with greater psychological improvements, these recommendations should be interpreted cautiously due to variability in study quality, intervention types, and sample characteristics.

## Data Availability

The original contributions presented in the study are included in the article/Supplementary Material, further inquiries can be directed to the corresponding author.

## Supplementary Materials

The following supporting information can be downloaded at: https://www.mdpi.com/article/10.3390/bs16010015/s1, Figure S1: Results of Cochrane risk of bias tool; Figure S2: Funnel plot (anxiety); Figure S3: Funnel plot (depression); Figure S4: Sensitivity analysis results (anxiety); Figure S5: Sensitivity analysis results (depression); Table S1: Search strategies; Table S2: Characteristics of the studies included in this meta-analysis; Table S3: Results of meta-regression; Table S4: Results of Egger’s test.

## Abbreviations

The following abbreviations are used in this manuscript:

T2DM	Type 2 diabetes mellitus
RCT	Randomized controlled trial
PRISMA	Preferred Reporting Items for Systematic Reviews and Meta-Analyses
MeSH	Medical Subject Headings
PICOS	Population, Intervention, Comparison, Outcome, Study design
SD	Standard deviation
RoB	Cochrane Risk of Bias
SE	Standard error
CI	Confidence interval
SMD	Standardized mean difference
BDNF	Brain-derived neurotrophic factor
IL-6	Interleukin-6
TNF-α	Tumor necrosis factor-alpha
ACSM	American College of Sports Medicine
CBT	Cognitive behavioral therapy
