# Supplementary material for: High-Frequency, Short-Session Exercise Decreases Anxiety and Depression in Individuals with Type 2 Diabetes Mellitus: A Systematic Review and Meta-Analysis of Randomized Controlled Trials
Source: Behav Sci (Basel). 2025 Dec 21;16(1):15. doi: 10.3390/bs16010015 (PMC12837154; doi:10.3390/bs16010015)
Supplement: Supplementary file 1 [file behavsci-16-00015-s001.zip › behavsci-4001280-supplementary.pdf]

Supplemental Material

Effects of exercise on depression and anxiety in type 2 diabetes mellitus: a systematic review and meta-analysis of randomized controlled trials

Figure S1. Results of Cochrane risk of bias tool. 2

Figure S2. Funnel plot (anxiety). 3

Figure S3. Funnel plot (depression)..4

Figure S4. Sensitivity analysis results (anxiety).......................................5

Figure S5. Sensitivity analysis results (depression). 6

Table S1. Search strategies. 7

Table S2. Characteristics of studies included in this meta-analysis. 8

Table S3. Results of meta-regression..11

Table S4. Results of Egger's test. 11

Figure S1. Results of Cochrane risk of bias tool.

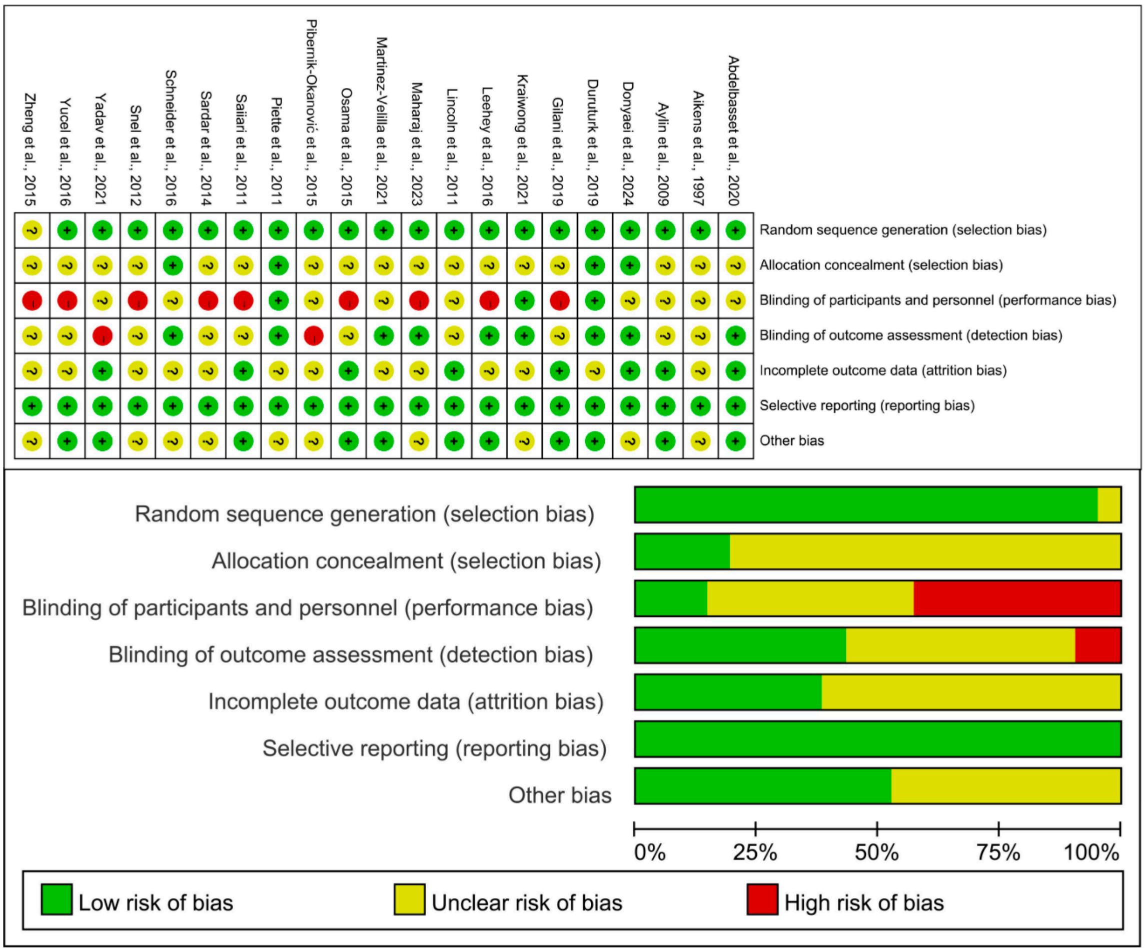

Figure S2. Funnel plot (anxiety).

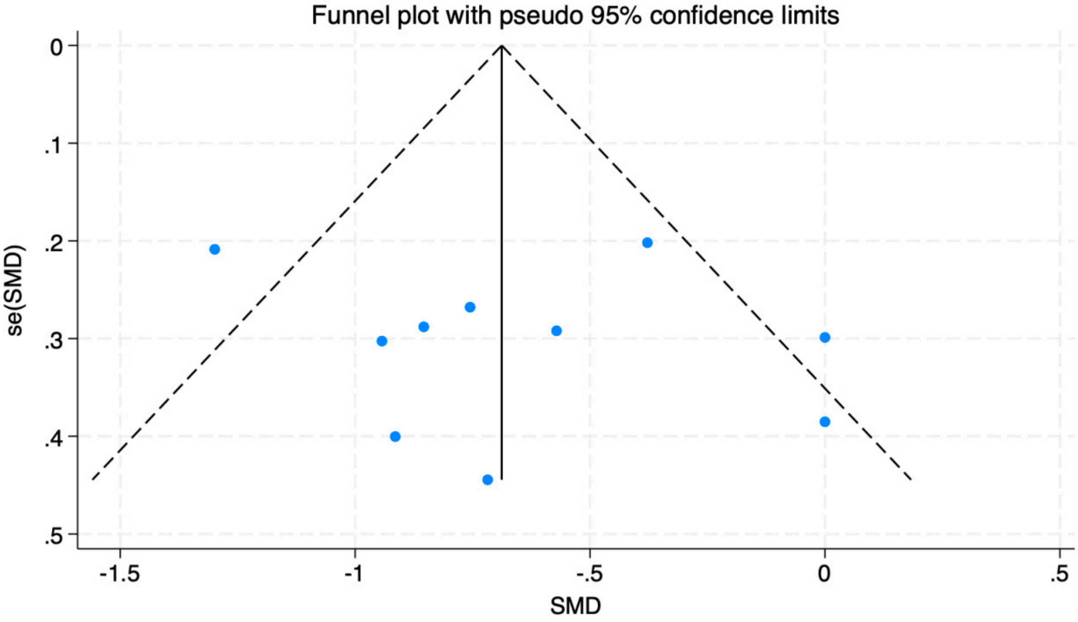

Figure S3. Funnel plot (depression).

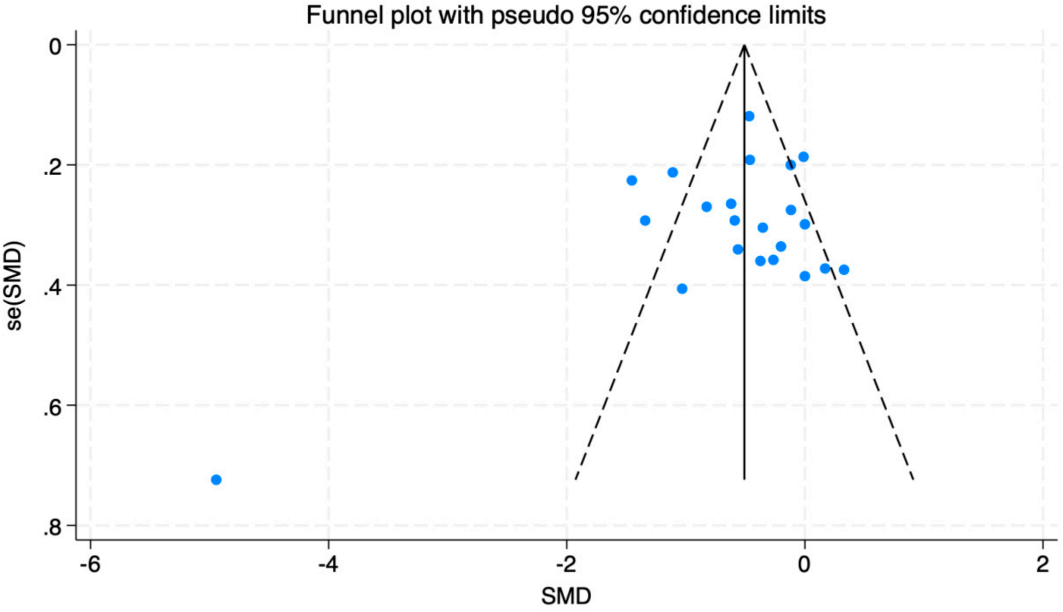

Figure S4. Sensitivity analysis results (anxiety).

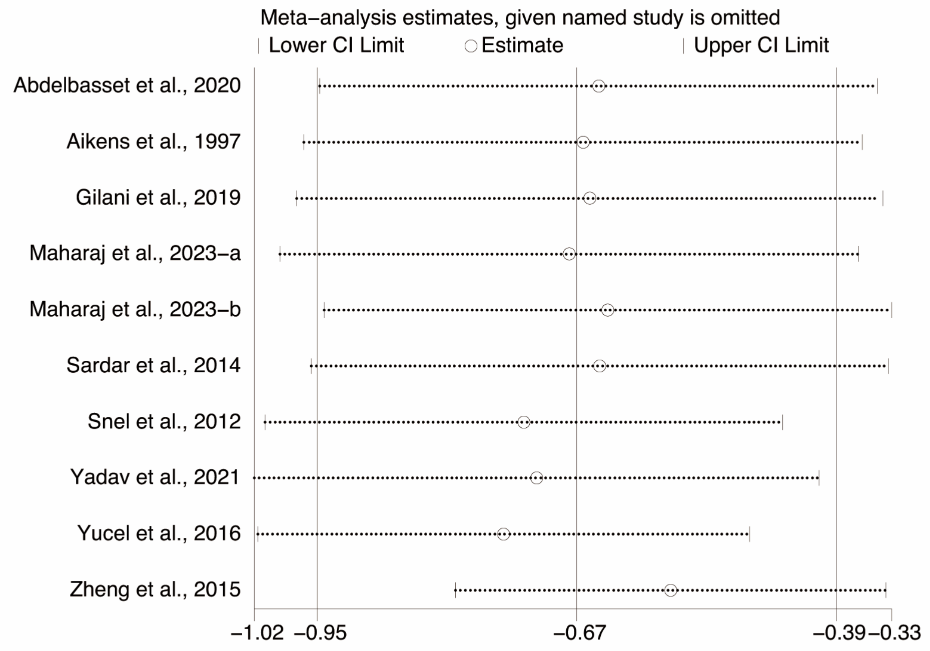

Figure S5. Sensitivity analysis results (depression).

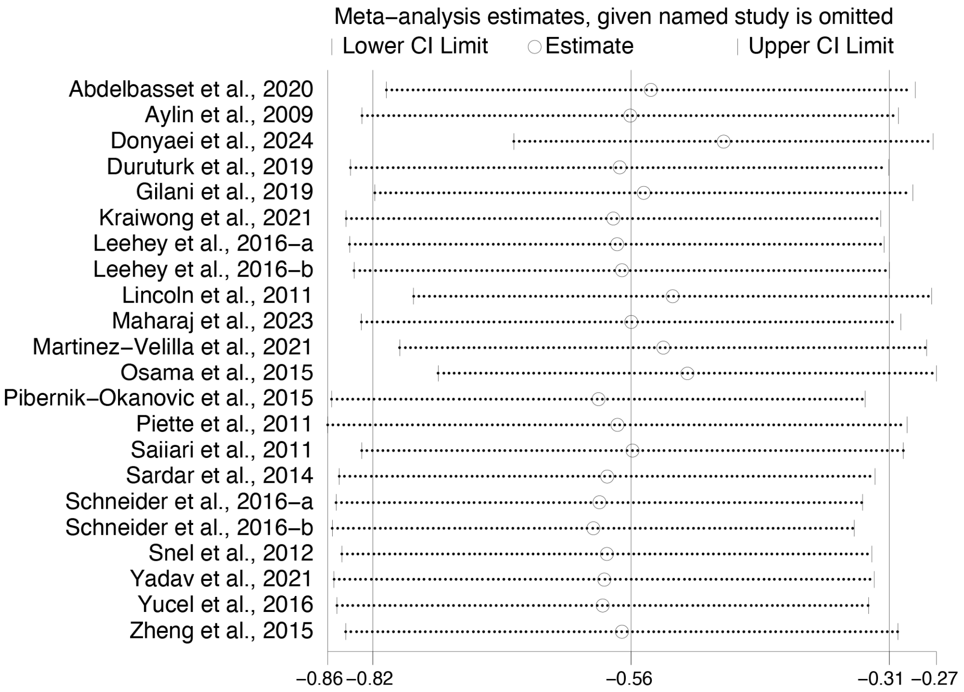

Table S1. Search strategies.

**Exercise search terms combined with "OR"**
Physical exercise programs; Physical Therapy Modalities; Physical Therapy Modalities; Modalities, Physical Therapy; Modality, Physical Therapy; Physical Therapy Modality; Physiotherapy; Physical Therapy Techniques; Physical Therapy Technique; Techniques, Physical Therapy; Exercise Movement Techniques; Exercise Movement Techniques; Movement Techniques, Exercise; Exercise Therapy; Exercise Therapy; Therapy, Exercise; Exercise Therapies; Therapies, Exercise; Exercise, Physical; Exercises, Physical; Physical Exercise; Physical Exercises; Exercise, Isometric; Exercises, Isometric; Isometric Exercises; Isometric Exercise; Exercise, Aerobic; Aerobic Exercises; Aerobic Exercise; Resistance Training; Resistance Training; Training, Resistance; Strength Training; Training, Strength; Weight-Lifting Strengthening Program; Strengthening Program, Weight-Lifting; Strengthening Programs, Weight-Lifting; Weight Lifting Strengthening Program; Weight-Lifting Strengthening Programs; Weight-Lifting Exercise Program; Exercise Program, Weight-Lifting; Exercise Programs, Weight-Lifting; Weight Lifting Exercise Program; Weight-Lifting Exercise Programs; Weight-Bearing Strengthening Program; Strengthening Program, Weight-Bearing; Strengthening Programs, Weight-Bearing; Weight Bearing Strengthening Program; Weight-Bearing Strengthening Programs; Weight-Bearing Exercise Program; Exercise Program, Weight-Bearing; Exercise Programs, Weight-Bearing; Weight Bearing Exercise Program; Weight-Bearing Exercise Programs
**Diabetes mellitus search terms combined with "OR"**
Diabetes; Diabetes Mellitus; Diabetes Insipidus; Diet, Diabetic; Prediabetic State; Scleredema Adultorum; Glycation End Products, Advanced; Glucose Intolerance; Gastroparesis
**Anxiety search terms combined with "OR"**
Anxiety; Angst; Nervousness; Hypervigilance; Social Anxiety; Anxieties, Social; Anxiety, Social; Social Anxieties; Anxiousness
**Depression search terms combined with "OR"**
Depression; Depressive Symptoms; Depressive Symptom; Symptom, Depressive; Emotional Depression; Depression, Emotional

Table S2. Characteristics of the studies included in this meta-analysis.

Study	Sample sizes	Age (y)	BMI (kg/m^2)	Intervention	Duration (weeks)	Frequency (times/week)	Minutes per session (min)	Depression outcomes	Anxiety outcomes
Abdelbasset et al., 2020	IG: 14<br>CG: 14	IG: 53.4 ± 5.3<br>CG: 52.8 ± 5.7	IG: 26.4 ± 2.8<br>CG: 27.2 ± 2.7	Proprioceptive exercise	8	3	45	BAI	HDRS
Aikens et al., 1997	IG: 12<br>CG: 10	61.0 ± 10.2	NA	Progressive muscle relaxation and imagery	6	1	60	GSI	NA
Aylin et al., 2009	IG: 18<br>CG: 18	IG: 51.39 ± 2.02<br>CG: 56.06 ± 1.48	IG: 28.45 ± 0.95<br>CG: 31.31 ± 1.12	RT and home based walking	8	4	90	NA	CES-D
Donyaei et al., 2024	IG: 17<br>CG: 17	IG: 61.3 ± 5.7<br>CG: 62.1 ± 5.1	IG: 30.2 ± 1.3<br>CG: 29.9 ± 1.2	RT, AT	12	3	60	NA	BDI
Duruturk et al., 2019	IG: 23<br>CG: 21	IG: 52.82 ± 11.86<br>CG: 53.04 ± 10.45	IG: 32.07 ± 6.51<br>CG: 29.90 ± 4.63	Breathing exercises and gymnastic activities	6	3	35	NA	BDI
Gilani et al., 2019	IG: 30<br>CG: 30	IG: 48.86 ± 5.76<br>CG: 49.08 ± 6.09	NA	AT	12	3	52.5	GHQ	GHQ
Kraiwong et al., 2021	IG: 22<br>CG: 15	IG: 70.09 ± 4.45<br>CG: 72.87 ± 6.42	IG: 24.44 ± 3.38<br>CG: 24.18 ± 3.78	AT, ST, BT	8	3	52.5	NA	PHQ-9
Leehey et al., 2016-A	IG: 14<br>CG: 18	IG: 65.4 ± 8.7<br>CG: 66.6 ± 7.5	IG: 36.2 ± 4.8<br>CG: 37.4 ± 4.2	RT, AT	12	3	90	NA	CES-D
Leehey et al., 2016-B	IG: 14<br>CG: 18	IG: 65.4 ± 8.7<br>CG: 66.6 ± 7.5	IG: 36.2 ± 4.8<br>CG: 37.4 ± 4.2	RT, AT	40	6	30	NA	CES-D
Lincoln et al., 2011	IG: 29<br>CG: 29	IG: 66.0 ± 7.9<br>CG: 66.6 ± 7.4	IG: 30.9 ± 5.7<br>CG: 31.2 ± 5.9	Progressive RT	16	3	45	NA	GDS

Maharaj et al., 2023	IG: 25<br>CG: 24	IG: 40.4 ± 7.22<br>CG: 39.9 ± 5.77	IG: 26.7 ± 4.96<br>CG: 27.5 ± 5.94	AT	12	3	45	STAI	BDI-II
Martinez-Velilla et al., 2021	IG: 54<br>CG: 49	IG: 87 ± 4<br>CG: 86 ± 5	IG: 27.7 ± 4.3<br>CG: 28.6 ± 5.2	Progressive resistance, balance, and walking exercises	12	12	20	NA	GDS
Osama et al., 2015	IG: 50<br>CG: 50	IG: 36.35 ± 5.11<br>CG: 37.16 ± 4.32	IG: 32.86 ± 5.29<br>CG: 33.15 ± 4.87	AT	12	3	40	NA	BDI
Pibernik-Okanović et al., 2015	IG: 58<br>CG: 57	NA	NA	PA	6	1	90	NA1	CES-D
Piette et al., 2011	IG: 145<br>CG: 146	IG: 55.1 ± 9.4<br>CG: 56.0 ± 10.9	IG: 37.3 ± 8.3<br>CG: 38.0 ± 9.3	CBT	48	NA	NA	NA	BDI
Saiiari et al., 2011	IG: 30<br>CG: 30	28 ~ 45	NA	AT	8	3.5	30	NA	BDI
Sardar et al., 2014	IG: 27<br>CG: 26	IG: 44.93 ± 5.35<br>CG: 45.56 ± 5.41	NA	AT	8	3	52.5	GHQ	GHQ
Schneider et al., 2016	IG: 15<br>CG: 14	IG: 53.3 ± 6.0<br>CG: 53.6 ± 8.4	IG: 34.5 ± 4.6<br>CG: 34.7 ± 6.0	Behavior activation and exercise	24	1.5	90	NA	BDI-II
Snel et al., 2012	IG: 13<br>CG: 14	IG: 53 ± 3<br>CG: 56 ± 2	IG: 36.4 ± 1.1<br>CG: 37.9 ± 1.4	AT	16	1	60	HADS	HADS

Yadav et al., 2021	IG: 50<br>CG: 50	IG: 52.24 ± 10.36<br>CG: 56.40 ± 11.72	NA	Training in diaphragmatic breathing and systematic relaxation	24	6	60	DASS-21	DASS-21
Yucel et al., 2016	IG: 24<br>CG: 21	IG: 58.50 ± 7.00<br>CG: 53.50 ± 9.00	IG: 32.20 ± 6.93<br>CG: 30.84 ± 8.09	PBME	12	3	57.5	HADS	HADS
Zheng et al., 2015	IG: 55<br>CG: 57	IG: 62 ± 6<br>CG: 61 ± 7	NA	24 type Tai Chi	24	8	40	SAS	SDS

Abbreviations: AT, aerobic training; BAI, the Beck Anxiety Inventory; BDI, the Beck's Depression Inventory; BDI-II, the Beck Depression Inventory II; BMI, body mass index; BT, balance training; CBT, telephone-delivered cognitive behavioral therapy; CES-D, the Centre for Epidemiological Studies-Depression; CG, control groups; DASS-21, the Depression, Anxiety, and Stress Scale–21; GDS, the Geriatric Depression Scale; GHQ, the General Health Questionnaire; GSI, the General Severity Index; HADS, the Hospital Anxiety Depression Scale; HDRS, the Hamilton Depression Rating Scale; IG, Intervention groups; NA, not available; PA, physical activity; PBME, pilates-based mat exercise; PHQ-9, the Patient Health Questionnaire-9; RT, resistance training; SAS, the Self-rating anxiety scale; SDS, the Self-rating depression scale; ST, strength training; STAI, the State-Trait Anxiety Inventory.

Table S3. Results of meta-regression.

Indicator	Modalities	ES	Coef.	Std. Err.	t	p > |t|	95% CI
Anxiety	Type	subgroup	-0.0083257	0.2938265	-0.03	0.978	-0.6858908, 0.6692394
		_cons	-0.6468321	0.4655499	-1.39	0.202	-1.720392, 0.4267279
	Frequency	subgroup	-0.3898222	0.397719	-0.98	0.356	-1.306964, 0.5273195
		_cons	0.063134	0.7522129	0.08	0.935	-1.671472, 1.79774
	Session duration	subgroup	0.4319059	0.2898691	1.49	0.175	-0.2365333, 1.100345
		_cons	-1.215238	0.3917382	-3.10	0.015	-2.118588, -0.311888
	Weekly time	subgroup	-0.2360429	0.326471	-0.72	0.490	-0.9888865, 0.5168007
		_cons	-0.3621605	0.4364625	-0.83	0.431	-1.368645, 0.6443239
Depression	Type	subgroup	0.1247818	0.4256358	0.29	0.773	-0.7660842, 1.015648
		_cons	-0.7455882	0.7515575	-0.99	0.334	-2.318616, 0.8274397
	Frequency	subgroup	-0.8363456	0.4563605	-1.83	0.083	-1.791519, 0.118828
		_cons	0.9501194	0.8466147	1.12	0.276	-0.8218655, 2.722104
	Session duration	subgroup	0.2138882	0.4168353	0.51	0.614	-0.6585581, 1.086335
		_cons	-0.8707765	0.6014326	-1.45	0.164	-2.129589, 0.3880364
	Weekly time	subgroup	0.1117714	0.5060719	0.22	0.828	-0.9474493, 1.170992
		_cons	-0.7184451	0.6425269	-1.12	0.277	-2.063269, 0.6263792

Abbreviations: Coef, coefficient; Std. Err, standard error; t, t-test statistic; CI, confidence interval.

Table S4. Results of Egger's test.

Indicator	Std_EFF	Coef.	Std. Err.	t	p > |t|	95% CI
Anxiety	Slope	-1.063841	0.5859793	-1.82	0.107	-2.415112, 0.28743
	Bias	1.371307	2.075088	0.66	0.527	-3.413855, 6.15647
Depression	Slope	-0.244227	0.3350741	-0.73	0.475	-0.9431794, 0.454725
	Bias	-1.114252	1.321428	-0.84	0.409	-3.870703, 1.642199

Abbreviations: Coef, coefficient; Std. Err, standard error; t, *t*-test statistic; p, probability; CI, confidence interval.
